# Glucosinolate biosynthesis: role of MAM synthase and its perspectives

**DOI:** 10.1042/BSR20211634

**Published:** 2021-10-04

**Authors:** Bidyadhar Das

**Affiliations:** Biological Chemistry Laboratory, Department of Zoology, School of Life Sciences, North-Eastern Hill University, Shillong 793022, India

**Keywords:** Arabidopsis thaliana, Brassicaceae plants, Glucosinolates, IPMS, MAM synthase

## Abstract

Glucosinolates, synthesized by the glucosinolate biosynthesis pathway, are the secondary metabolites used as a defence mechanism in the Brassicaceae plants, including *Arabidopsis thaliana*. The first committed step in the pathway, catalysed by methylthioalkylmalate (MAM) synthase (EC: 2.3.3.17), is to produce different variants of glucosinolates. Phylogenetic analyses suggest that possibly MAM synthases have been evolved from isopropylmalate synthase (IPMS) by the substitutions of five amino acid residues (L143I, H167L, S216G, N250G and P252G) in the active site of IPMS due to point mutations. Considering the importance of MAM synthase in Brassicaceae plants, Petersen et al. (2019) made an effort to characterise the MAM synthase (15 MAM1 variants) *in vitro* by single substitution or double substitutions. In their study, the authors have expressed the variants in *Escherichia coli* and analysed the amino acids in the cultures of *E. coli in vivo*. Since modifying the MAM synthases by transgenic approaches could increase the resistance of Brassicaceae plants for enhancing the defence effect of glucosinolates and their degraded products; hence, MAM synthases should be characterized in detail *in vivo* in *A. thaliana* along with the structural analysis of the enzyme for meaningful impact and for its imminent use *in vivo*.

## Introduction

**Figure 1:**
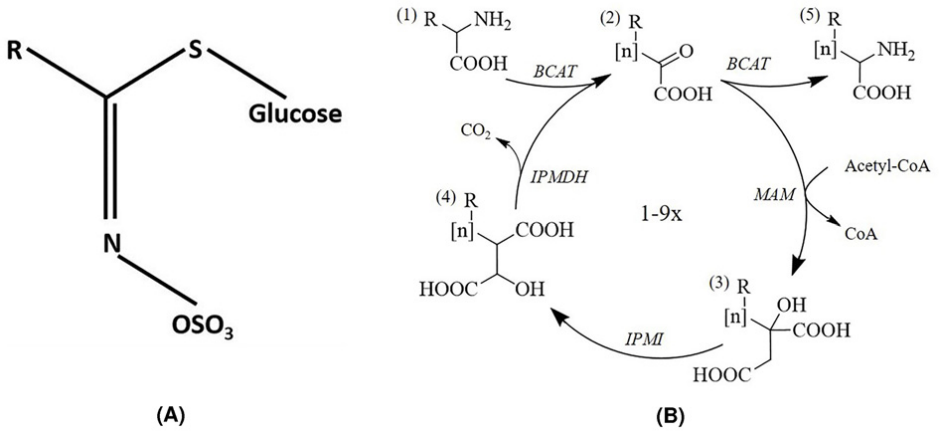
Glucosinolate biosynthesis pathway in Brassicaceae plants (**A**) Typical structure of glucosinolate [8]. (**B**) Excerpts from Petersen et al. [7] showing a schematic view of the role of MAM in the chain elongation in glucosinolate biosynthesis pathway. The first step is a deamination reaction catalysed by a branched-chain aminotransferase, followed by a three-step cycle reaction catalysed by three enzymes, MAM, isopropyl malate isomerase and isopropyl malate dehydrogenase.

Glucosinolates, derived from the amino acids (like alanine, leucine, isoleucine, methionine, phenylalanine, tryptophan, tyrosine and valine), are the secondary metabolites used as a defence mechanism (with the help of the enzyme thioglucosidase myrosinase) in the Brassicaceae plants (like cauliflower, cabbage, broccoli), including *Arabidopsis thaliana*. Glucosinolates also play an important role in human health as an anti-cancer and antimicrobial agent. These secondary metabolites are generally found within the vacuoles of specialized cells of these plants. The glucosinolate (Figure 1A) contains a thio-glucose moiety, a sulfonated oxime, and a side-chain “R” derived from amino acids. The “R” group undergoes numerous chain elongations (mainly from methionine) before the core particle of glucosinolate is synthesized by the glucosinolate biosynthesis pathway (Figure 1B).

Generally, the pathway consists of three steps: (1) amino acid chain-elongation, (2) core structure formation and (3) secondary modifications. The chain elongation step catalysed by the enzyme methylthioalkylmalate (MAM) synthase (EC: 2.3.3.17) is very important and the first committed step in this pathway to produce different variants of glucosinolates. This reaction determines the “R” group length of the glucosinolates. There are about 60 homologues of MAM synthases found in the Brassicaceae plants.

## Evolution of MAM synthase

MAM synthase catalyses the leucine biosynthesis in bacteria, fungi and plants; however, it catalyses the chain elongation in methionine-derived glucosinolate biosynthesis in Brassicaceae. Phylogenetic analyses suggest that MAM synthases are derived from isopropylmalate synthase (IPMS), which catalyse the initial reaction of leucine biosynthesis [1]. Another phylogenetic analysis of plant IPMS homologues supports the hypothesis that the Brassicaceae IPMS gives rise to MAM synthases [2]. Based upon phylogenetic analyses and overlap of catalytic sites, it seems MAM synthases are most likely evolved from IPMS through a process of gene duplication and change in enzyme function [3]. There are two novel properties found in MAM synthases: (1) substitutions of amino acid residues in the active site of MAM synthases due to point mutations, which has changed substrate specificity and utilize methylthio-derivatives of IPMS substrate (2-oxoisovalerate) and (2) absence of the C-terminal domain in MAM synthases responsible for leucine end-product inhibition in IPMS enzymes [1].

## Structural analysis of the MAM synthase

A structural homology search recognizes IPMS, citramalate synthase, and homocitrate synthase (HCS) as the closest structural relatives of MAM1-A of *Brassica juncea* [4]. Comparison of the monomer structures of MAM synthase and IPMS reveals that the overall fold of MAM synthase shares the N-terminal catalytic α/β-barrel domain and the C-terminal α-helical region that forms part of the coenzyme A binding site with IPMS. The major structural dissimilarity between MAM synthase and IPMS includes an additional C-terminal domain that permits for feedback inhibition by leucine in IPMS and an extension of the N-terminal in IPMS, which both are lacking in MAM synthase [1,4].

IPMS and MAM synthases sequences of Arabidopsis share approximately 60% amino acid similarity. It has been observed that in MAM synthase, five amino acids, L143, H167, S216, N250 and P252, differ from the catalytic domain of IPMS. These amino acid residues are altered by substitutions, L143I, H167L, S216G, N250G and P252G, in MAM1 synthase or MAM3 synthase; serine-to-glycine substitution (S216G) being the first replacement in MAM synthase from IPMS. The second substitution is a proline in IPMS to glycine in MAM synthase (P252G). Besides the replacement of S216G and P252G in MAM synthase from IPMS, few other amino acids substitutions in IPMS2/R2 resulted in inactive enzymes. Apart from H167, these amino acid residues are located at the C-terminal ends of the β-strands or within the loops that link β-strands with α-helices. Despite general product profiles assessment, key point mutations as well as alterations in the N- and C- terminal domains modify IPMS toward the evolution of MAM synthase [1].

## Substitution of amino acid residues dictates the substrate specificity

**Figure 2:**
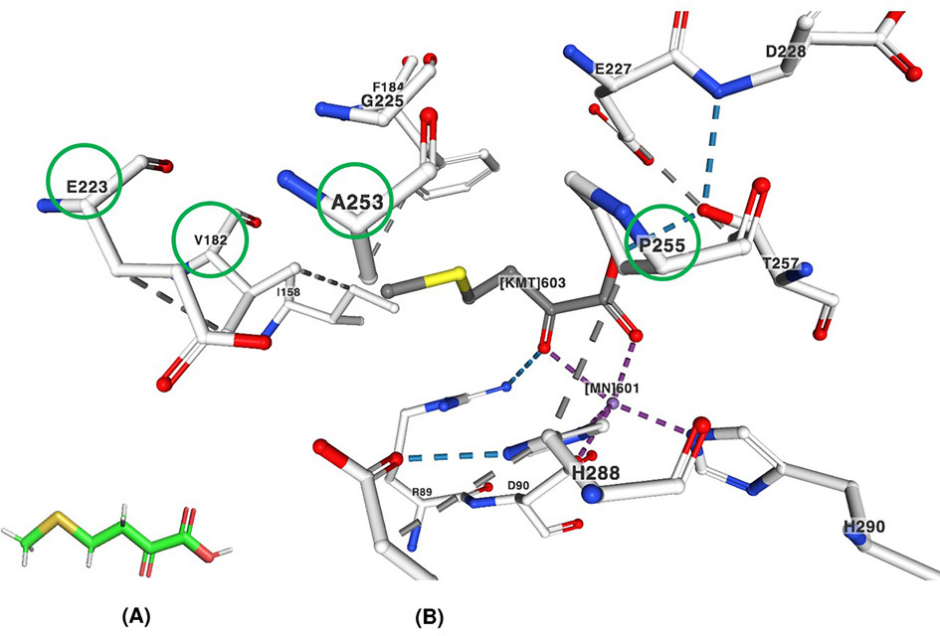
Interactions of amino acid residues with the substrate of MAM synthase from Brassica juncea (**A**) 3D format of the substrate 4-methyl-thio-2-oxobutanoic acid. (**B**) Interactions of the encircled amino acid residues (V182, E233, A253 and P255) with the substrate 4-methyl-thio-2-oxobutanoic acid (labelled as KMT603) and Mn^2+^ and other amino acid residues around the active site of BjuMAM1.1 (PDB ID: 6E1J, MAM synthase from *Brassica juncea*) [4]. The bonds are represented by different colours: hydrogen bonds (blue), hydrophobic contacts (grey) and metal interactions (purple), respectively.

The active site of MAM synthase is located on the C-terminal face of the α/β-barrel domain [4]. The replacement of amino acid residues in the active site of MAM with glycine might be expected to fit larger substrates, the derivatives of glucosinolate. Serine-to-glycine substitution (S216G) has been noted to have a greater effect on introducing MAM synthase activity into IPMS [1]. Analysis of the MAM synthase crystal structure (PDB ID: 6E1J) and sequence comparisons of the four *B. juncea* MAM synthase recognize four amino acid residues that differ between the two sets of isoforms. The crystal structure (PDB ID: 6E1J) complexed with 4-methyl-thio-2-oxobutanoic acid and Mn^2+^ shows that the amino acid residues (V182, E233, A253 and P255) interact with the substrate 4-methyl-thio-2-oxobutanoic acid in BjuMAM1.1 (Figure 2), MAM synthase from *Brassica juncea* [4]. These amino acids are substituted by V182L, G223Q, A253N and P255A, respectively, in the MAM2 proteins. These four amino acid residues also form a major portion of the substrate-binding site and likely govern the substrate preference of the enzyme MAM synthase [4]. Multiple MAM synthase genes are found in *the A. thaliana* genome and biochemical studies show that the formation of short-chain (C3-C5) aliphatic glucosinolates is catalysed by MAM1 synthase and MAM2 synthase, whereas MAM3 synthase catalyses the formation of short and long-chain (C6-C8) glucosinolates [4].

The changes in the protein sequences explain the evolution of MAM synthase in *A. thaliana* from IPMS [1]. The expression patterns of glucosinolate side chain biosynthetic genes MAM1 and MAM3 of *A. thaliana* are different in different vegetative parts and developmental stages [5]. The overexpression of three glucosinolate biosynthesis genes in brassica napus enhances resistance to *Sclerotinia sclerotiorum* and *Botrytis cinerea* by transgenic approaches or molecular breeding [6]. Methionine-derived aliphatic glucosinolates catalysed by MAM synthase can provide an evolutionary and biochemical foundation for the diversification of glucosinolate [4].

## Evaluation of the manuscript

**Figure 3:**
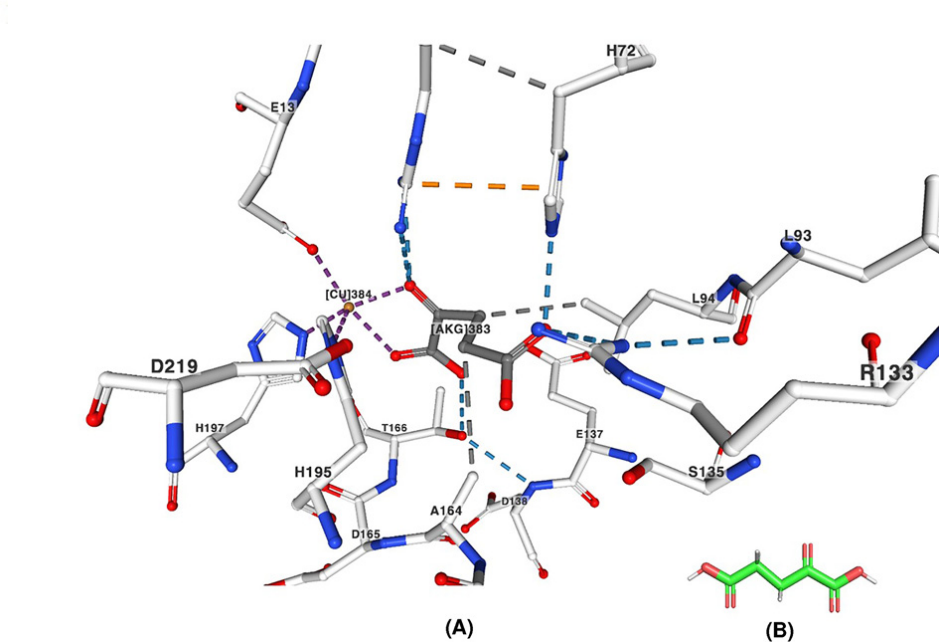
Interactions of amino acid residues with the substrate of HCS from Thermus thermophilus (**A**) Interactions of the amino acid residues with the substrate 2-oxoglutarate (labelled as AKG383) and other amino acid residues around the active site of the HCS from *Thermus thermophilus* [9] complexed with alpha-ketoglutarate (PDB ID: 2ZTJ). (**B**) 3D format of the substrate 2-oxoglutarate. The bonds are represented by different colours: hydrogen bonds (blue), hydrophobic contacts (grey), Pi interactions (orange) and metal interactions (purple), respectively.

In the manuscript [7], an attempt has been made to characterise the MAM enzymes (15 MAM1 variants by single substitution or double substitutions and the native MAM1 and MAM3 from *A. thaliana*) by heterologous expression in *Escherichia coli*. In their study, the authors have identified four polymorphic amino acid residues, which interact with the substrate (2-oxo acid), based on the alignment of 57 MAM sequences and protein structure modelling comparison of MAM1 from *A. thaliana* with the crystal structure of HCS (EC 2.3.3.14; PDB ID: 2ZTJ). Figure 3 shows the interaction of the amino acid residues with the substrate 2-oxoglutarate in the active site of HCS. The following observations have been made [7].

## Protein structure modelling of MAM enzyme

**Figure 4:**
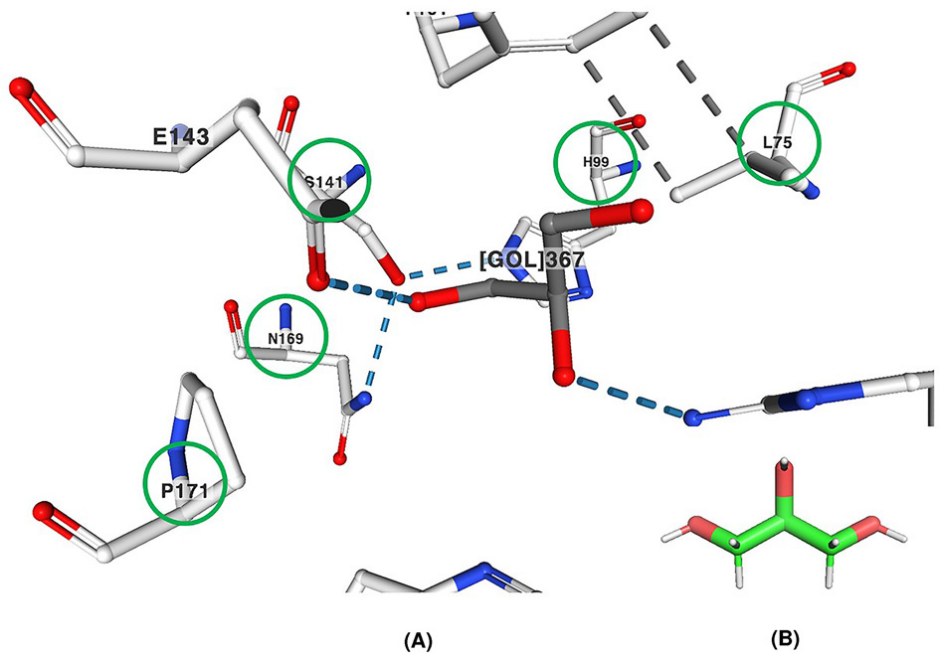
Interactions of amino acid residues with the substrate of IPMS from Neisseria meningitidisi (**A**) Interactions of the encircled amino acid residues (L75, H99, S141, N169 and P171) with the substrate glycerol (labelled as GOL367) and other amino acid residues around the active site of the truncated IPMS from *Neisseria meningitidisi* [10] complexed with glycerol (PDB ID: 3RMJ). (**B)** 3D format of the glycerol. The bonds are represented by different colours: hydrogen bonds (blue) and hydrophobic contacts (grey).

As mentioned in the manuscript, to date there is no MAM synthase crystal available from *A. thaliana* in Protein Data Bank; hence, the authors prepared the homology model of the enzyme MAM1 synthase structure using the template of IPMS (EC 2.3.3.13; PDB ID: 3RMJ) and superimposed. The interaction of the amino acid residues (L75, H99, S141, N169 and P171) with the substrate glycerol in the active site of IPMS is shown in Figure 4. The authors assumed that in MAM1 synthase the substrate, α-ketoglutarate, binds similarly as in the crystal structure of HCS (PDB ID: 2ZTJ); hence, the amino acid residues (L186, T257 and G259 and A290) were predicted for MAM1 synthase active site within 8 Å radius of the substrate binding.

However, it would be more accurate, if the atomic coordination comparative analyses had been done between the HCS crystal (PDB ID: 2ZTJ) and the MAM1 synthase homology model docked with α-ketoglutarate. This would have shown the networking of the amino acid residues, types of bonds, and distance between the atoms in the active site of MAM1 synthase. In this study, based upon the assumption, the authors have identified four reactive amino acid residues interactions, out of which the only A290 has not been described in detail. This study is a bioinformatic analysis of similar enzymes, hence, it is difficult to predict the exact amino acid residues involved in the enzymatic reaction of MAM1 synthase.

The active site of an enzyme determines its enzyme kinetics, therefore, the authors should confirm that homology modelling of MAM1 is good enough at determining the overall three-dimensional folds of the enzyme. Most of the time it suffers great inaccuracy when determining the side chain geometries, which are essential in the determination of the active sites of enzymes. In this study, the authors have not taken care of the side chain geometries of the enzyme while preparing the homology model of the enzyme MAM1. Further, regarding the homology modelling, which may not be a good predictor of the finer details of protein structure (particularly side-chain positioning of amino acid residues or position of the rotamers), the authors should provide more accurate details regarding the MAM1 homology model. The computational tools (like validation and minimization) might minimize the steric conflicts and alter the side chain geometries and the rotamer conformations, to achieve the orientations of amino acids that are typically populated. Therefore, these kinds of analyses, while preparing the homology model, achieve minimum energy for the global homology structure of MAM1.

Volume Area Dihedral Angle Reporter would have been useful for quantitative protein structure evaluation. The correctness of these minimized structures may vary greatly when compared to the authentic structure in various liganded states, as used by the authors in this study (PDB ID: 2ZTJ). The authors should be aware that it is typical that energetically favourable contacts, made during the folding, are used to offset energetic penalties to achieve the unique side-chain and main-chain positioning in enzymes in creating the active site of an enzyme. These are necessary for the enzyme kinetics and specific function of the enzyme. Thus, I would reiterate that homology modelling is an excellent tool for discerning the overall three-dimensional folds of an unknown protein when an accurate template is used and the side chain geometries of the enzyme are taken care of. But, using a homology modelled structure as the input for the functional sequel is not an ideal statement.

## Metabolite analysis of the chain elongation pathway with MAM synthase variants

The amino acid levels in the cultures of *E. coli* transformed with different MAM synthase variants have been monitored and compared with the levels of *E. coli* strain transformed with the empty vector. It has been found that the cultures of *E. coli* transformed with different MAM synthase variants contained more phenylalanine (about 3 times higher) and leucine (about 7 times higher) than methionine. The liquid chromatography-mass spectrometry analysis of the cultures of *E. coli* has shown the expression of the chain elongation pathway with different MAM synthase variants. These experiments have been performed in a heterologous system *E. coli*, hence, it would be interesting to note whether these chain elongations are replicated in *A. thaliana* or not. Therefore, in my opinion, the experiments need to be performed in vivo in *A. thaliana* for evaluation of the variants.

## Analysis of MAM1 and MAM3 mutants for homophenylalanine (HPhe)-derived glucosinolate

Analysis of both MAM1 and MAM3 of Arabidopsis for Hphe-derived glucosinolate has been found that MAM1 produced high HPhe (20-fold higher) than MAM3. Glucosinolate analysis in MAM1 and MAM3 mutants of *A. thaliana* shows MAM1 is responsible for Hphe-derived glucosinolate production. This analysis has been done in heterologous expression in *E. coli*, hence, these experiments need to be performed in *A. thaliana* to replicate the observations and its functional analysis.

## Protein expression assessed by targeted proteomics

The expression level of the proteins of the chain elongation enzymes, expressed in *E. coli*, has been monitored by targeted proteomics of proteotypic peptides representing the individual proteins. Though the authors have shown in this study that various differential expression level of protein expression was expressed harbouring different substitutions, however, it would be better to analyse the expression level of the proteins and their corresponding mRNAs using Western blots and Real-Time polymerase chain reaction, respectively.

## Conclusion

In conclusion, in addition to the present published work, more accurate bioinformatic details are required for the MAM1 homology model. The molecular interactions of the substrate with the MAM variants *A. thaliana* should be determined by using X-ray crystallography or cryo-electron microscopy. Further, the enzyme kinetics and circular dichroism analysis of the MAM variants of *A. thaliana* should be performed spectrophotometrically concerning Michaelis–Menten constants for the substrate and other co-factors. Finally, the functional consequences of the amino acid substitution (both single and double substitutes) in the MAM variants should be analysed *in vivo*, particularly in *A. thaliana*. Transgenic approaches might increase the resistance of Brassicaceae plants for enhancing the defence effect of glucosinolates and their degraded products. In the published manuscript [7], the authors have expressed the variants in *E. coli* and analysed the amino acids in the cultures of *E. coli in vivo*. In my opinion, MAM synthases should be characterised in detail *in vivo* in *A. thaliana* along with the structural analysis of the enzyme for meaningful impact and for its imminent use *in vivo*.

## Abbreviations

HCS: homocitrate synthase
HPhe: homophenylalanine
IMPS: isopropylmalate synthase
MAM: methylthioalkylmalate
